# Preparticipation screening—the way forward is smart screening

**DOI:** 10.1007/s12471-018-1081-9

**Published:** 2018-02-02

**Authors:** H. Thune Jørstad

**Affiliations:** Fellow Sports Cardiology, Department of Cardiology, Academic Medical Center—University of Amsterdam, Amsterdam, The Netherlands

**Keywords:** Sports cardiology, Preparticipation screening, Sudden cardiac death, Sudden cardiac arrest

## Abstract

The image of a young athlete collapsing on the pitch, followed by resuscitation, leaves an unforgettable impression. However, this impression should not seduce us into resuscitating the debate for large-scale preparticipation screening without doing the smart thing: taking a step back to review what we know to be effective, and what has been shown not to be effective. What we *should* do is use this momentum to focus on what we still *need to know*.

**Figure:**
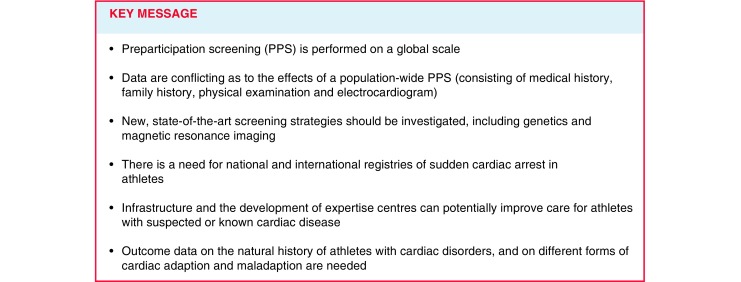

If an image or impression can be remembered, it must be important, or in any case, more important than the things we do not remember. This is called the ‘availability heuristic’, and was described by Tversky and Kahneman in 1973. Before this, the prevailing school of thought was that humans were exclusively rational actors within the field of judgement under uncertainty, utilising extensive mental processing to arrive at a decision. Opposed to this school of thought, Tversky and Kahneman postulated that judgment under uncertainty relies on a limited number of simplifying heuristics, one of which is the availability heuristic. In short, what we remember, must be important. Few things trigger this heuristic more effectively than the image of an athlete, the symbol of health and youth, collapsing on the pitch. Invariably, the ensuing debate segues into questions as to how this could have been prevented, and voices are raised for comprehensive preparticipation screening (PPS) programmes. However, medical professionals should be aware of the availability heuristic and its influence on public opinion. In addition, it would be unfortunate if other players in this field, such as insurance companies, politicians and policymakers, overreact in the absence of medical expertise, and make decisions that profoundly affect both medical professionals and athletes.

Why is the availability heuristic important? Because it offers a plausible explanation for the fact that sudden cardiac death (SCD) in young competitive athletes, although rare, has a tremendous impact. While quantification and registration of such events leaves much to be desired, reported incidences vary from 1:917,000 to 1:3,000 athlete-years, with an estimated incidence of 1:50,000 athlete-years [1]. Yet, unlike many other rare diseases, sudden cardiac death in athletes has sparked a diversity of initiatives and intense debate on how to prevent it, even taking into account that no screening programme can prevent SCD completely. When the now famous Veneto data were published, it seemed that a comprehensive PPS, including electrocardiography, would be the way forward [2]. In short, after the implementation of a nationwide PPS in Italy, there was evidence of a dramatic decline (89%) in sudden cardiac death in young competitive athletes compared with the period before the screening, with a concordant increase in the identification and disqualification of athletes with cardiac abnormalities, in particular cardiomyopathies, found during PPS. However, these results were not replicated elsewhere [3], and countries without mandatory screening (e. g. US, France) reported similar decreases in the incidence of sudden cardiac death [4, 5].

Following this, Van Brabandt et al. (2016) published a meta-analysis strongly cautioning against the implementation of nationwide screening programmes, arguing poor detection rates, potential harm due to false positives, and negative psychological and financial consequences [6]. One might expect that these findings would immediately lead to a decrease in mandatory PPS, but there appears to be an increase in the number of screenings as opposed to the expected decrease, although hard numbers are lacking [7]. One important reason might be that it is almost impossible to defend *not screening* athletes when the pictures of an athlete collapsing on the pitch can go viral and reach millions on the internet within a few hours, facilitated by a medium famous for its lack of balanced discussion, scientific or otherwise, in its mainstream outlets. How do we explain false positive and false negative in 140 characters?

Do the conflicting data regarding PPS mean that we should give up on screening altogether? The plethora of existing screening programmes gives a strong indication that we are already past this point. Most screening programmes have been focused on methods to screen as many athletes as possible with the lowest possible costs and effort. Instead of discussing the restart of a population-wide screening programme in the Netherlands, we should be discussing ways to develop a *smart screening programme*. In an extensive registry study in Canada, 80% of all out-of-hospital cardiac arrests in competitive athletes would not have been prevented with conventional PPS [8]. Therefore, one way forward may be to screen those athletes who train and perform at the highest intensity, and have the highest visibility. Instead of aiming to screen the largest possible number of competitive athletes, a more intensive screening of a smaller number of athletes, with state-of-the-art diagnostic tools, such as magnetic resonance imaging (MRI) and genetics, within a structure of expert centres, could potentially not only be a smarter way to reduce the number of rare catastrophes, but could also greatly contribute to our understanding of athletes’ cardiac adaptation and conditions.

In the Netherlands, different organisations require participants to undergo heterogeneous screening initiatives, performed by different organisations and physicians. Such screenings are reimbursed by some, but not all insurance policies, dependent on the level of coverage and the insurance company. There is currently no national consensus on who should be screened, how the screening should be performed, and how it should be financed [7]. Due to the conflicting data, a wide variety of strategies may be implemented in different settings and sports. Therefore, now is the time for medical professionals to engage in defining the role of screening and designing appropriate diagnostic strategies.

First, the medical professionals should urge policymakers and insurers to sponsor high-quality research into the cardiac consequences of extreme amounts of training and performance in athletes. In line with this, the American Medical Society for Sports Medicine (AMSSM) recently issued a statement in which they caution against the universal use of PPS, but also identify several knowledge gaps to stimulate the initiation of research programmes in this field [9]. One of their recommendations is the development of national, mandatory registries (including autopsy data) of athletes with sudden cardiac death or aborted sudden cardiac death. It has been suggested that athletes performing certain sports (e. g. basketball) are at a higher risk, and that risks differ between ethnicities, but clear data are lacking [1]. The AMSSM further recommends establishing an infrastructure for local collaborations and partnerships between sports physicians and sports cardiologists. Other proposals are the creation of regional referral centres to assist in electrocardiogram interpretation and evaluation of athletes with suspected or known cardiovascular disorders [10]. Another knowledge gap concerns the paucity of outcome data focusing on the natural history of athletes with cardiac disorders, to improve risk stratification with continued sports participation and to increase our understanding of the cardiovascular effects, in health and disease, of physical exercise in different types of sports. Currently, there is a limited amount of data concerning the cardiac effects of (extreme) training, and how we can identify the individuals who are at the highest risk of adverse events. There is still no clear-cut answer to questions such as, what are the optimal levels and volumes of training for continuous cardiac improvement? and, which levels are potentially dangerous? Is there such a thing as ‘cardiac overtraining’, can it be predicted, quantified, and/or prevented? There are currently no evidence-based answers.

During the annual Laurence H. Green memorial lecture at the Brigham and Women’s Hospital (Boston, Massachusetts), the director of the American National Heart, Lung, and Blood Institute (NHLBI), Gary H. Gibbons, used a young athlete-as-patient case to illustrate his envisioned ‘virtuous’ cycle of research for the coming years. To support the development of precision medicine, the NHLBI envisions a new taxonomy that accurately describes pathophysiology and disease (and separate this from adaptive changes) and incorporates different modalities, such as molecular diagnostics, imaging, and analytics, in addition to more ‘conventional’ modalities. The NHLBI wants to achieve this by creating a cycle of basic, translational and clinical research, including, but not limited to, prospective cohorts, clinical studies, machine learning, and genetic studies. When precision medicine allows reliable identification of athletes at risk for sudden cardiac death, large-scale screening may be considered. Furthermore, improved risk quantification in athletes should enable athletes and their trainers to decrease risk through tailored training programmes. Shared decision-making approaches should also allow athletes to understand the consequences of and make decisions, insofar possible, about further participation in competitive sports. For medical professionals, the time is now to stimulate a greater research effort. The tragic events that take place on the pitch should, in the end, motivate all those involved to contribute to our own virtuous cycle of research, with the same zeal and ambition as the competitive athlete.
